# surtvep: An R package for estimating time-varying effects

**DOI:** 10.21105/joss.05688

**Published:** 2024-06-28

**Authors:** Lingfeng Luo, Wenbo Wu, Jeremy M. G. Taylor, Jian Kang, Michael J. Kleinsasser, Kevin He

**Affiliations:** 1 Department of Biostatistics, School of Public Health, University of Michigan; 2 Departments of Population Health and Medicine, New York University Grossman School of Medicine

## Abstract

The surtvep package is an open-source software designed for estimating time-varying effects in survival analysis using the Cox non-proportional hazards model in R. With the rapid increase in large-scale time-to-event data from national disease registries, detecting and accounting for time-varying effects in medical studies have become crucial. Current software solutions often face computational issues such as memory limitations when handling large datasets. Furthermore, modeling time-varying effects for time-to-event data can be challenging due to small at-risk sets and numerical instability near the end of the follow-up period. surtvep addresses these challenges by implementing a computationally efficient Kronecker product-based proximal algorithm, supporting both unstratified and stratified models. The package also incorporates P-spline and smoothing spline penalties to improve estimation (Eilers & Marx, 1996). Cross-validation and information criteria are available to determine the optimal tuning parameters. Parallel computation is enabled to further enhance computational efficiency. A variety of operating characteristics are provided, including estimated time-varying effects, confidence intervals, hypothesis testing, and estimated hazard functions and survival probabilities. The surtvep package thus offers a comprehensive and flexible solution to analyzing large-scale time-to-event data with dynamic effect trajectories.

## Statement of Need

The Cox non-proportional hazards model is a flexible and powerful tool for modeling time-varying effects of covariates in survival analysis. However, as the size of a dataset increases, the computational costs of this model can become substantial. Current software solutions, which may be effective for smaller datasets, face challenges when handling larger datasets.

Numerous studies have demonstrated the widespread presence of time-varying effects. For instance, the scientific literature has shown that factors like age, sex, and race can have non-constant associations with survival in cases such as end-stage renal disease (He et al., 2017, 2022), and breast cancer patients receiving neo-adjuvant chemotherapy and head and neck cancer patients (Baulies et al., 2015; Brouwer et al., 2020). Ignoring the variations and relying solely on the Cox proportional hazards model can lead to inaccurate risk prediction and suboptimal treatment development.

With the rising need for modeling time-varying effects, researchers have developed methods to handle the complex and dynamic nature of such data (Gray, 1992, 1994; Hastie & Tibshirani, 1993; Zucker & Karr, 1990). In terms of implementation, these methods expand the original data in a repeated measurement format (Therneau et al., 2017) using existing software such as the survival package (Therneau, 2023). Even with moderate sample sizes, this leads to a large and computationally burdensome working dataset. surtvep addresses this issue by implementing a computationally efficient Kronecker product-based proximal algorithm (Perperoglou et al., 2006), which can handle time-varying effects in large-scale studies with improved efficiency and parallel computing capabilities. Compared with existing computational packages for Cox non-proportional hazards models, such as the coxph function, surtvep demonstrates a much more efficient performance, with both runtime and memory consumption reduced considerably.

Another issue of numerical instability arises when analyzing data with binary covariates that have limited variation. surtvep implements a proximal Newton’s method to improve the estimation. Additionally, adding a penalty can improve the estimation. surtvep also supports P-spline and smoothing spline (Eilers & Marx, 1996; Wood, 2017a, 2017b), to further improve estimation stability. The improved estimation performance of surtvep is demonstrated in our recent studies (Luo et al., 2023; Wu et al., 2022).

**Figure 1:**
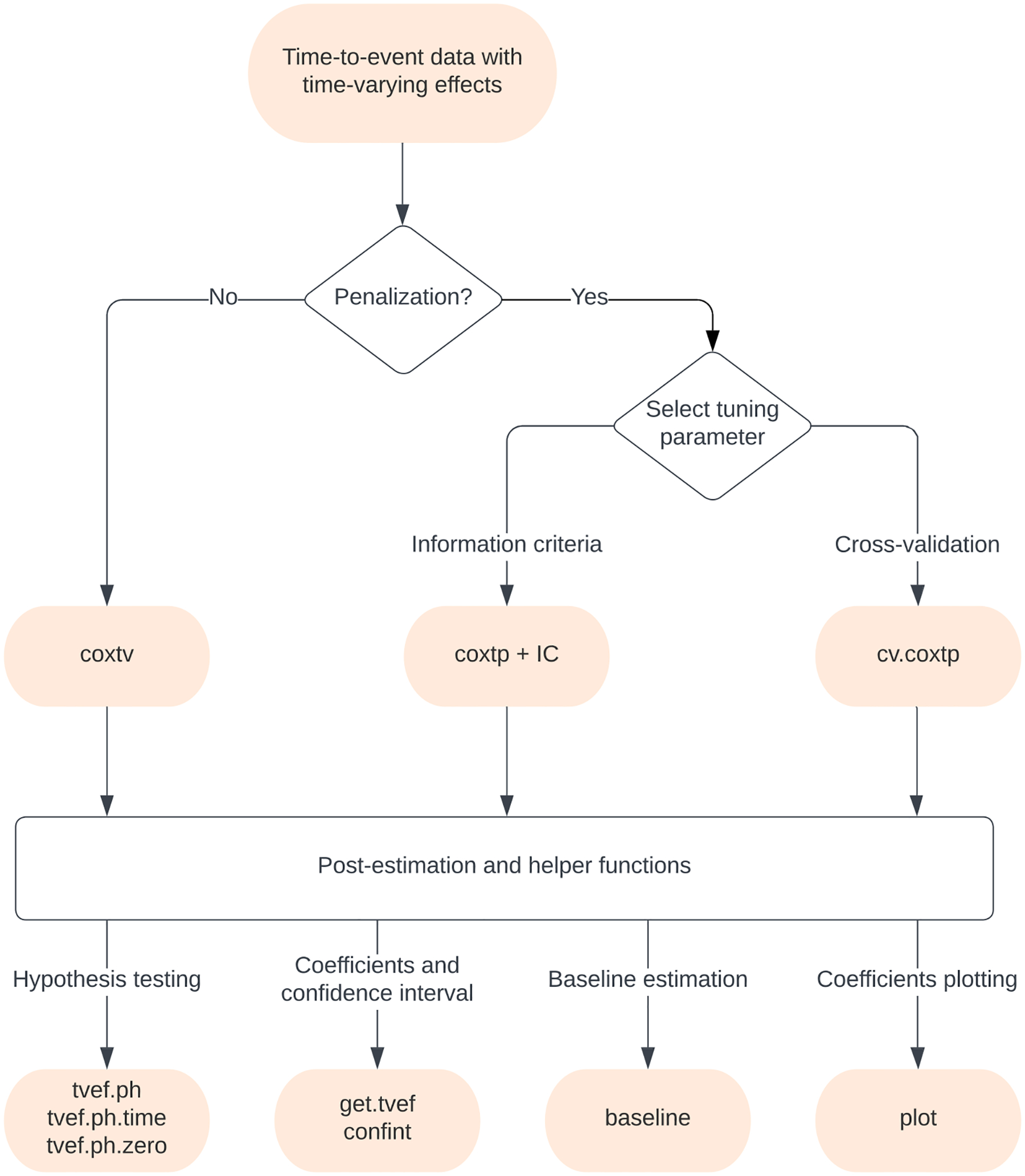
Flowchart for functions in the surtvep package. coxtv utilizes proximal Newton’s method to estimate the time-varying coefficients. coxtp combines the Newton’s approach with penalization. IC calculates different information criteria to select the best tuning parameter in front of the penalty term. cv.coxtp uses cross-validation for tuning parameter selection. tvef.ph, tvef.ph.time and tvef.ph.zero provide hypothesis testing for the fitted model. get.tvef retrieves the time-varying coefficients for the fitted model. confint provides confidence intervals for these coefficients. baseline offers the baseline hazard estimations. plot visualizes the estimated time-varying coefficients.

Finally, our method has several other features worth noting. First, surtvep supports the stratified model, which enables researchers to account for differences in baseline hazard functions across distinct clusters or other grouping factors. This is particularly useful when there are distinct subgroups within the data that may have different baseline hazards. Second, surtvep enables shared-memory parallel computation features, which can significantly improve the performance of the software when working with large datasets. Also, surtvep supports Breslow approximation (Breslow, 1974), which significantly improves the computational speed when a large number of ties are present. The functions and workflow of the surtvep package are summarized in the flowchart in Figure 1.

## Functions

surtvep is a powerful statistical software package designed for analyzing time-varying effects of time-to-event data. The software offers two main functions for estimating time-varying coefficients in survival analysis.

To model time-varying coefficients in surtvep, we first define the time-varying coefficients as *β*(*t*), which represents the effects of predictors at different time points. We then use a set of B-spline basis functions to span the *β*(*t*), which provides a flexible and accurate way to capture the time-dependent effects of the predictors. These B-spline basis functions are generated using the splines R package with a fixed number of basis functions. While the effect of the predictors vary with time, the predictors are assumed to have a linear relationship with the outcome.

Once we have established the basis functions for the time-varying coefficients, coxtv employs a proximal Newton’s approach to estimate the coefficients in front of the B-spline basis functions. This approach iteratively updates the coefficients until a maximum of the log-partial likelihood is reached. Backtracking line search is utilized to improve the estimation. We have also implemented a shared-memory parallelization to enable faster convergence.

coxtp is the second main function, adding a penalty term to the original objective function. This approach iteratively updates the coefficients until a maximum of the penalized log-partial likelihood is reached. coxtp provides two options for penalized regression: P-spline and smoothing spline.

- P-spline stands for penalized B-spline. It combines the B-spline basis with a discrete quadratic penalty on the difference of basis coefficients between adjacent knots. When the penalty term goes to infinity, the time-varying effects are reduced to be constant.
- Smoothing spline is a derivative-based penalty combined with B-spline. When the cubic B-spline is used for constructing the basis functions, the smoothing spline penalizes the second-order derivative, which reduces the time-varying effect to a linear term when the penalty term goes to infinity. When the quadratic B-spline is used for constructing the basis functions, the smoothing spline penalizes the first-order derivative, which reduces the time-varying effect to a constant when the penalty term goes to infinity. See Wood (2017b) for details.

surtvep also provides a function IC to select the best tuning parameter in front of the penalty term. IC can be used to calculate the modified Akaike information criterion (mAIC), the Takeuchi information criterion (TIC) and the generalized information criterion (GIC) (Akaike, 1998; Luo et al., 2023; Takeuchi, 1976). Generally, mAIC, TIC and GIC have relatively similar performance. Using one of these criteria to select tuning parameters is considerably faster than using cross-validation, which is also provided in surtvep via function cv.coxtp.

Finally, surtvep offers a comprehensive suite of hypothesis testing capabilities, allowing researchers to assess the validity and significance of their models (Wu et al., 2022). Specifically, surtvep can perform the following hypothesis tests: (1) testing the proportional hazards assumption to verify the model’s suitability for the given data; and (2) examining the pointwise significance of covariate effects at different event times to assess the impact of each covariate on the outcome of interest. To conduct these hypothesis tests, surtvep employs the Wald test statistic, a widely-used method for inference.

## Quick Start

The purpose of this section is to introduce the basics of surtvep. Interested users are referred to the online tutorial at https://um-kevinhe.github.io/surtvep/articles/surtvep.html for detailed instructions.

surtvep can be easily installed by launching an R prompt and running the following commands:


install.packages(‘surtvep’)
library(surtvep)


Next, we load an example data set that includes two columns z of continuous covariates, a column time indicating the time to an event, and a column “event” of event indicators.


data(“ExampleData”)
z <- ExampleData$z
time <- ExampleData$time
event <- ExampleData$event


We can fit the Newton’s method without penalization using the most basic call to coxtv. For the Newton’s method with penalization, we call the coxtp function.


fit.tv <- coxtv(z = z, event = event, time = time)
fit.penalize <- coxtp(z = z, event = event, time = time)


We use IC to calculate the information criteria and select the best tuning parameter:


fit.ic <- IC(fit.penalize)


fit.tv is an object of class coxtv that contains all the relevant information of the fitted model for further use. fit.ic contains three objects of class coxtp, corresponding to the selected model using mAIC, TIC and GIC. Various methods are provided for the objects such as plotting and hypothesis testing.

**Figure 2:**
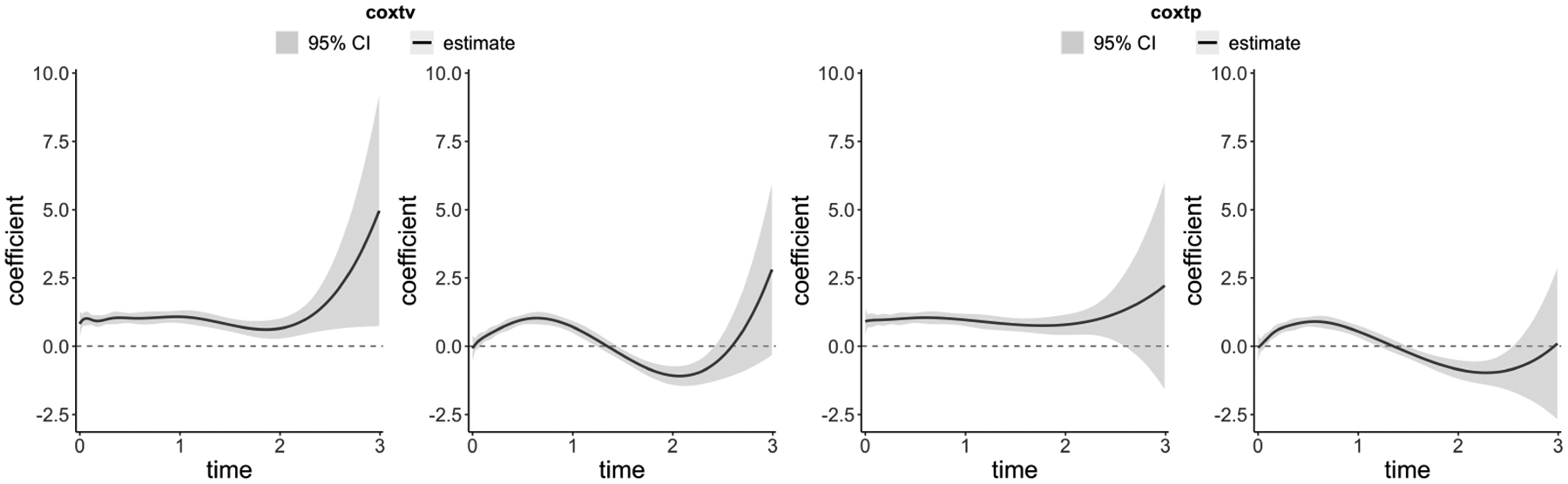
The estimated time-varying coefficients (log hazard ratio) from coxtv and coxtp. The tuning parameter for coxtp is selected using mAIC.

The code below generates Figure 2, which visualizes the time-varying coefficients from coxtv and coxtp:


plot(fit.tv, ylim = c(−3,10))
plot(fit.ic$model.mAIC, ylim = c(−3,10))


In utilizing the coxtv and coxtp functions, users have the flexibility to choose based on their dataset’s specifications. Numerical instabilities are commonly encountered when analyzing survival data of a small sample size or when the data includes some binary covariates with proportions that approach either zero or one. In these scenarios, the second-order information matrix can become ill-conditioned. See discussion in (Luo et al., 2023; Wu et al., 2022). To address this issue, the employment of the penalized method coxtp is recommended. When determining the number of basis functions, a typical range is between 5–10. Though the choice is somewhat flexible, it has limited impact on results unless set too small (Gray, 1992). Users might consider increasing this number when applying the penalized method.

Currently, both coxtv and coxtp assume time-varying effects for all covariates. We primarily focus on low-dimensional settings (where the number of covariates is much smaller than the sample size) and support only right-censored survival data. Future releases will expand these capabilities.

## Data Example

**Figure 3:**
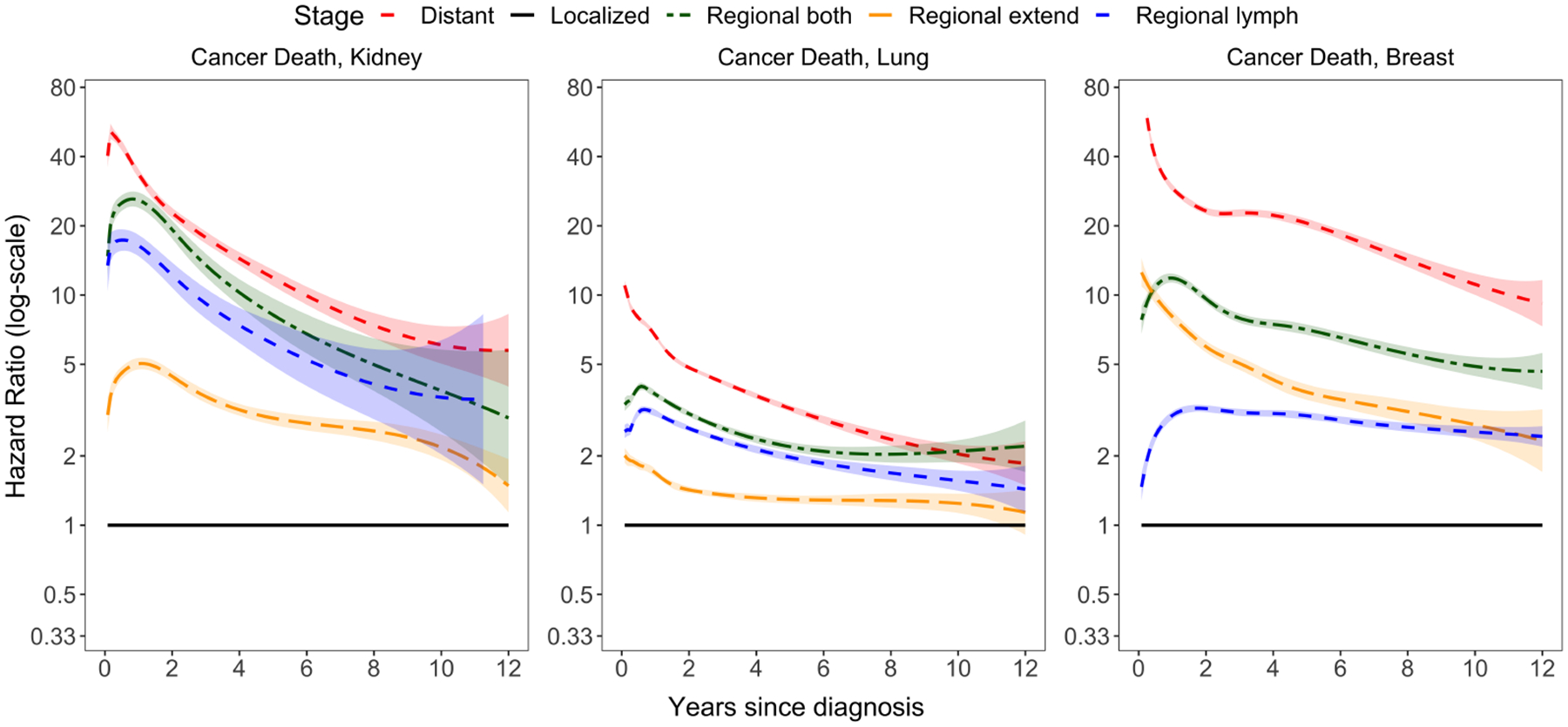
Time-varying effects of cancer stage in SEER data.

We demonstrate the effectiveness of surtvep by applying it to a real-world dataset from the National Cancer Institute Surveillance, Epidemiology, and End Results (SEER) Program (National Cancer Institute, 2019). We estimate the hazard ratios of the cancer stage of kidney, lung, and breast, as shown in Figure 3. Our analysis highlights the dynamic nature of hazard ratios for cancer death among patients with metastatic stage compared to those with localized stage. Access to SEER data can be requested at https://seer.cancer.gov/data/access.html for those interested.

In the first year after diagnosis, the hazard ratio is strikingly high, indicating a significant difference in survival outcomes between metastatic and localized stage patients. However, this disparity shrinks considerably by the eighth year, reflecting the diminishing relevance of the initial cancer stage in the prognosis of long-term survivors. This example illustrates the importance of accounting for time-varying effects, which has been effectively addressed by surtvep through its flexible and efficient approach to modeling these dynamics. By providing accurate and efficient modeling of time-varying effects in large-scale datasets, surtvep serves as a valuable tool for researchers working with complex survival data.

## Data Availability

Stable releases of the surtvep package will be made available via the Comprehensive R Archive Network. Alternatively, the surtvep package is available on GitHub (https://github.com/UM-KevinHe/surtvep). Use of the surtvep package has been extensively documented in the package documentation and on the tutorial website (https://um-kevinhe.github.io/surtvep/index.html).
